# Audio-Based Characterization of Gait Parameters in Mangalarga Marchador, Campolina, and Piquira Horses Using Deep Learning

**DOI:** 10.3390/ani16091283

**Published:** 2026-04-22

**Authors:** Alan Freire, Alisson Vitor da Silva, Laura Patterson Rosa, Paulo Henrique Sales Guimarães, Brennda Paula Gonçalves Araujo, Carlos Augusto Freitas Silva, Larissa Raffaela Trindade Borges, Antônio Gilberto Bertechini, Sarah Laguna Conceição Meirelles

**Affiliations:** 1 Faculdade de Zootecnia e Medicina Veterinária, Universidade Federal de Lavras Campus Universitário, Lavras 2720-000, MG, Brazil; alissonzoovet@gmail.com (A.V.d.S.); brennda.pga95@gmail.com (B.P.G.A.); carlos.silva29@estudante.ufla.br (C.A.F.S.); larissaraffaela@gmail.com (L.R.T.B.); bertechini@ufla.br (A.G.B.); 2 Department of Veterinary Clinical Sciences, Lewyt College of Veterinary Medicine, Long Island University, Brookville, NY 11542, USA; laura.patterson@liu.edu; 3 Instituto de Ciências Exatas e Tecnológicas, Campus Universitário, Lavras 2720-000, MG, Brazil; paulo.guimaraes@ufla.br

**Keywords:** dissociated gait, equine locomotion, artificial intelligence, neural networks

## Abstract

The evaluation of biomechanical parameters in four-beat gaited horses remains limited by the subjectiveness and complexity of current standard methods. Through a deep learning approach, we aimed to infer dissociation % using only acoustic signals. A total of 268 audio samples were extracted from publicly available videos featuring three Brazilian horse breeds (Mangalarga Marchador, Campolina, and Piquira) performing marcha batida and marcha picada. Acoustic features, including root mean square energy (RMS), zero-crossing rate (ZCR), and 13 Mel-frequency cepstral coefficients (MFCCs), were extracted and used to train a long short-term memory (LSTM) neural network. The model accurately predicted the time intervals between successive hoof–ground contacts (R^2^ = 0.98; MAE = 0.0071), enabling the calculation of the dissociation %. While no significant differences were found between gait types and dissociation %, breed-related differences in both mean hoof–ground contact interval and dissociation were observed, with 8 acoustic features demonstrating discriminative power. Our results suggest that hoof–ground contact patterns can be quantified objectively from audio alone, offering a practical and non-invasive method for gait analysis. The approach holds potential for applications in breed standardization, selection, and digital locomotion phenotyping of horse populations.

## Simple Summary

The dissociation percentage between limbs during locomotion in gaited horses is a critical variable associated with comfort and gait regularity, yet it is still assessed subjectively during official evaluations. In this study, we expanded the application of an existing non-invasive method to predict the degree of dissociation using only the sound emitted during horse locomotion, testing it on a subpopulation of gaited horses. A long short-term memory (LSTM) neural network was trained using acoustic features extracted from videos of Brazilian gaited horse breeds—Mangalarga Marchador, Campolina, and Piquira—performing marcha batida and marcha picada. The model achieved high accuracy (R^2^ = 0.98) in predicting the time intervals between hoof–ground contacts and allowed for the calculation of dissociation (%). No significant differences were found between gait types for dissociation, yet breed-specific differences and unique audio features were observed. Our findings suggest that acoustic data can serve as a reliable source for biomechanical gait analysis and may support future tools for performance evaluation, selection, and digital phenotyping in equine breeding programs.

## 1. Introduction

Gait evaluation in horses still relies primarily on the evaluator’s auditory and visual perception, limiting accuracy and consistency in assessing key biomechanical parameters underlying locomotor quality and functional performance, which are critical for selection and genetic improvement programs [1,2,3]. This subjectivity is particularly relevant for Brazilian gaited breeds (Mangalarga Marchador, Campolina, and Piquira) and donkeys (Jumento Pêga) known for performing a spectrum of four-beat intermediate gaits. The breeders’ associations, through their respective breed standards, characterize these gaits by the absence of suspension phases and a four-beat, dissociated limb support sequence. Dynamically, these consist of alternating advances of forelimbs and hindlimbs, with a predominance of either diagonal (marcha batida) or lateral (marcha picada) bipedal supports, intercalated by moments of tripodal support. These locomotion patterns enhance rider stability and are highly valued, and the dissociation between individual limbs is a key parameter in official judging events and in the selection of breeding animals [4,5,6,7,8]. However, despite their economic and cultural importance, gait evaluation in these animals remains largely dependent on human interpretation, introducing bias and inconsistency into scoring systems used for selection and genetic improvement.

Among the biomechanical features that define these intermediate four-beat gaits, the degree of dissociation, defined as the temporal separation between successive hoof–ground contacts, is directly related to limb coordination and movement efficiency and commonly associated with rider-perceived comfort and gait quality [9]. In official competitions promoted by the breeders’ associations, gait evaluation relies on the subjective visual and auditory perception of hoof–ground contact sequences [10,11]. This approach depends on the experience of the evaluator and is subject to bias and sensory limitations and can demonstrate inconsistency among judges at official competitions and breed inspectors [12,13]. As competition outcomes directly influence the commercial value of the animals and guide selection and breeding decisions, the lack of standardization in gait assessment represents a technical challenge. Therefore, there is an urgent need for objective, low-cost, and easily applicable methods for consistent and standardized gait evaluation, both in competitions and on farms.

Over the past three decades, several technologies have been developed for objective equine gait assessment, including force plates, pressure walkways, high-speed kinematic video analysis, and inertial measurement units (IMUs) [14,15]. These methods have contributed to a deeper understanding of locomotor biomechanics and lameness detection, yet their application in routine field conditions and official competitions remains limited due to cost, equipment requirements, and environmental constraints. This gap highlights the need for practical and low-cost strategies capable of standardizing gait analysis outside of the laboratory setting. In parallel, a growing body of research in humans has explored acoustic signals as proxies for gait dynamics. Acoustic gait studies have demonstrated that footstep sounds vary systematically with fatigue levels, footwear and flooring, and even subtle differences in strike patterns, revealing the potential of sound as a non-invasive indicator of biomechanical properties [16,17,18,19]. However, studies also underscore methodological challenges: acoustic signatures can differ depending on microphone type, sensor placement, and environmental conditions, with issues such as latency and noise strongly influencing signal interpretation [20,21]. Thus, while acoustic approaches are promising, they must be applied with careful consideration of these limitations.

Acoustic research in animals has expanded in recent years, and the extraction of spectro-temporal features such as Mel-frequency cepstral coefficients (MFCCs), root mean square energy (RMSE), and zero-crossing rate (ZCR) [22,23] has demonstrated effectiveness in various sound classification tasks. These features have shown notable performance: achieving up to 94% accuracy in detecting pig wasting diseases from vocalizations [24], reliably discriminating between different animal species in wildlife monitoring contexts [25], and detecting foraging behavior in horses using sound patterns associated with chewing [26]. Despite these advances, to our knowledge, no studies were performed with the goal to predict continuous biomechanical parameters related to degree of dissociation based exclusively on the sound captured during equid gait in four-beat locomotion patterns. A single, pioneering work has used acoustic data to distinguish between gait types (marcha batida and marcha picada) in Brazilian gaited horses [27].

We hypothesize that the temporal structure of hoof–ground impact sounds contains sufficient information to predict the degree of dissociation and limb coordination patterns in gaited horses, enabling the transformation of auditory judgment into a high-resolution quantitative metric. Thus, our goal was to develop and validate a neural network pipeline based on the Long Short-Term Memory (LSTM) architecture, capable of quantifying both the degree of dissociation and the interval between hoof strikes in three distinct Brazilian horse breeds, the Mangalarga Marchador, Campolina, and Piquira, using exclusively acoustic data. This approach could provide an objective, non-invasive, and low-cost tool to support functional assessment, technical training, and future genetic improvement of Brazilian gaited horse breeds.

## 2. Materials and Methods

### 2.1. Ethical Approval

No direct handling, intervention, or experimentation involving live animals was conducted during the research, and data was exclusively obtained from publicly available audio files; thus, an Ethics Committee approval was not required. The analysis was performed using audio signal processing and deep learning techniques applied to pre-existing recordings, ensuring compliance with ethical guidelines for non-invasive studies.

### 2.2. Data Collection

The data used in this study was obtained from publicly available videos on the YouTube platform of three Brazilian horse breeds: Mangalarga Marchador (MM), Campolina (CAM), and Piquira (PQ). For each of these breeds, 12 videos were selected featuring the marcha picada and 12 videos with the marcha batida, totaling 72 videos. The selection process was carried out by a previously trained evaluator with technical experience in gait classification, who categorized the animals according to breed and type of gait based on the official standards of the registry associations governing each breed [1,2,3]. All selected videos contained more than two complete gait cycles (5–8 strides per cycle, depending on gait type and breed) to ensure sufficient time coverage for parameter estimation. We extracted audio tracks from these videos to be utilized as the main data source for acoustic analysis, capturing the sounds of the hoof’s impact with the ground during locomotion.

All animals included in the analysis were estimated to be older than 36 months, as evidenced by being ridden under saddle with gait proficiency consistent with mature individuals of these breeds. In the selected videos, horses were filmed moving in a straight line, which reduced potential gait asymmetries associated with uneven locomotion paths. Of the 72 individuals, 58 were observed on concrete or paved surfaces, while the remaining 14 were moving on compacted dirt ground. The exact type of horseshoe could not be determined from the recordings; however, frame-by-frame visual inspection indicated that 65 of 72 horses were shod with metal shoes.

To limit the inclusion of unsound animals, videos were independently screened by a veterinarian co-author (LPR) using visual gait assessment criteria recommended in the American Association of Equine Practitioners (AAEP) lameness scale. Horses displaying head nodding, pelvic asymmetry, shortened strides, toe dragging, overt limb irregularities, or other visual indicators consistent with pain or lameness were excluded from analysis. No horse included in the final dataset demonstrated visible signs of discomfort or lameness.

### 2.3. Audio Processing, Filtering, and Cleaning

All procedures, including audio pre-processing, feature extraction, and model development, were performed using Python 3.12 in the Google Colab environment (Google LLC., Mountain View, CA, USA). The following libraries were used: librosa (v0.10.1) for audio processing and MFCC extraction, scipy (v1.11.4) for band-pass filtering, noisereduce (v2.0.1) for noise suppression, tensorflow.keras (v2.15.0) for construction and training of the LSTM model, and scikit-learn (v1.4.0) for cross-validation and evaluation metrics. Data manipulation and visualization were carried out using numpy, pandas, and matplotlib. All dependencies were installed within the Collab environment using pip. The source code was executed on a virtual machine equipped with an NVIDIA Tesla T4 GPU (16 GB VRAM) and 12 GB RAM (Google Cloud, Google LLC., Mountain View, CA, USA).

We extracted the audio from all videos in .wav format using the free online tool Y2DOWN (https://y2down.cc), with PCM encoding at 44.1 kHz. After extraction, the audio files underwent a preprocessing procedure to ensure and standardize the acoustic quality of the audio used in the analysis by removing unwanted sounds, such as human voices and environmental noise, which could interfere with the accurate identification of gait patterns, as follows:

Initially, a band-pass filter implemented using the SciPy library [28], a fifth-order Butterworth band-pass filter, was applied using the scipy.signal.butter() function, followed by bidirectional filtering with scipy.signal.filtfilt() to prevent phase distortion. This approach was implemented directly in the time domain, which is suitable for temporally compact signals such as acoustic waveforms. We defined the cutoff frequency range between 100 Hz and 2000 Hz, guided by short-time Fourier transform (STFT) analyses with a 2048-sample window, which revealed that hoof–ground impact events consistently exhibited dominant spectral energy between 200 and 1250 Hz. In our dataset, relevant harmonic components extended up to approximately 1800 Hz. The 100–2000 Hz window was therefore selected to preserve the transient energy associated with hoof strikes while attenuating low-frequency environmental noise and high-frequency components unrelated to gait events. Similar frequency windows have been recommended in previous studies for isolating footfall impacts in animal locomotion signals [29,30]. To confirm the effectiveness of the filtering process, we computed and analyzed spectrograms before and after filtering using STFT. The raw signals showed broad-spectrum content with overlapping noise and irrelevant harmonics. After filtering, the spectrograms revealed clear preservation of the acoustic transients within the selected frequency band, confirming the filter’s selectivity and adequacy.

Despite the application of the band-pass filter, small residual noises were still present in the audio signals. To further refine the signal quality, a noise reduction procedure was implemented using the NoiseReduce library. Specifically, we applied the nr.reduce_noise() function, which performs adaptive spectral gating by estimating the noise profile based on segments of low energy and subtracting it from the signal without affecting transient acoustic events. The audio files were processed using the following configuration: parameter stationary = True, applying a constant noise threshold, acting as a uniform suppressor of low-energy components, in order to preserve the acoustic transients associated with hoof impacts, avoiding the distortion that could occur with an adaptive mask.

We also retained the default value of n_fft = 2048, providing a spectral resolution of approximately 46 ms (at 44.1 kHz), suitable for capturing fast transients. This step was applied after the band-pass filtering to further refine the signal by attenuating residual background noise without compromising the events of interest [28,31].

### 2.4. Data Augmentation

To enhance the robustness of the deep learning model and improve its generalization capability, a data augmentation process was applied to the audio files [32]. This procedure involved generating artificial variations from the original files, creating 268 new samples, thereby expanding the dataset used for training. To increase dataset diversity and improve model generalization, four data augmentation techniques were systematically applied to the original audio files. First, pitch shifting was performed by varying the pitch of each audio sample by ±2 semitones, preserving the temporal structure while altering frequency content. Second, controlled white noise was added to simulate realistic background disturbances, enhancing the model’s robustness to environmental noise. Third, time stretching was applied to adjust the speed of the audio signal by ±10%, mimicking natural fluctuations in gait rhythm. Finally, time shifting was used to displace the entire audio waveform along the temporal axis, allowing the model to learn consistent patterns despite changes in the timing of hoof–ground contact events [33,34]. These transformations contributed to increasing dataset diversity, enhancing the model’s ability to recognize different gaits, even in the presence of subtle acoustic variations. All augmented audio files for Campolina, Mangalarga Marchador, and Piquira horses are available in the Supplementary Materials (Table S2).

### 2.5. Feature Extraction

After preprocessing and cleaning, we performed a feature extraction to identify relevant characteristics for the analysis of equine gaits, using the Python Librosa library [35], capturing acoustic information that describes the properties of the sounds emitted during the animals’ movement [36]. Audio features were extracted using functions from the Librosa library. The root mean square energy (RMS) was computed using the function librosa.feature.rms, with the input signal stored in the variable y, which represents the audio waveform loaded using librosa.load. The resulting values were assigned to the variable rms. The zero crossing rate (ZCR) was extracted using librosa.feature.zero_crossing_rate, also applied to the variable y, and the output was stored in the variable zcr. This metric provides information about the frequency of zero-crossings in the signal, which is relevant for detecting rhythmic hoof–ground contact patterns [37]. The Mel-frequency cepstral coefficients (MFCCs) were extracted using the function librosa.feature.mfcc, configured with n_mfcc = 13, and the output was saved in the variable mfccs. These coefficients capture the spectral envelope of the sound using perceptually meaningful frequency bands [38]. Finally, all extracted features (rms, zcr, and mfccs) were organized into a list of dictionaries stored in the variable features_list. This list was then converted into a structured dataset using a Pandas DataFrame [39] and stored in the variable features_df. Finally, the dataset was exported to a .csv file using the command features_df.to_csv() for later use in model training and evaluation. The complete dataset of extracted features used in this study is provided in the Supplementary Materials (Table S1).

### 2.6. Deep Learning Model Construction

The architecture implemented in this study is based on a long short-term memory (LSTM) deep neural network, widely recognized for its efficiency in time-series analysis, such as the time intervals between hoof support phases. This choice is justified by LSTM’s ability to capture temporal patterns in sequential data, preserving contextual information over time [38,40]. The model was designed with a specific configuration to optimize performance for the proposed task. Initially, an LSTM layer with 128 units and ReLU activation was employed, followed by a dropout layer with a 30% rate to mitigate the risk of overfitting. Next, a second LSTM layer with 64 units was added, complemented by a dense layer with 32 neurons, also using ReLU activation. Finally, the architecture was concluded with an output layer consisting of a single neuron, responsible for continuously predicting time intervals [41].

The training process was conducted over 100 epochs using the mean squared error (MSE) [42] as the loss function. This metric calculates the average of the squared differences between the predicted values and the actual target values in the training set. In this context, $y_{i}$ represents the true average time interval between hoof beats for sample i, while ${\hat{y}}_{i}$ denotes the corresponding predicted value generated by the model.

In the Adam optimizer [43,44], the hyperparameters $\beta_{1}$ and $\beta_{2}$ control the exponential decay rates for the moving averages of the gradients and the squared gradients, respectively. Typically, $\beta_{1}$ is set to 0.9 and determines how much past gradients influence the current estimate (momentum effect), while $\beta_{2}$, often set to 0.999, governs how quickly the optimizer adapts to changes in the gradient’s magnitude. These parameters help stabilize learning and accelerate convergence, especially in noisy or sparse gradient environments where $g_{t}$ is the gradient of the loss function at time step t, and are the hyperparameters controlling the decay rates [43,44].

**Figure 1:**
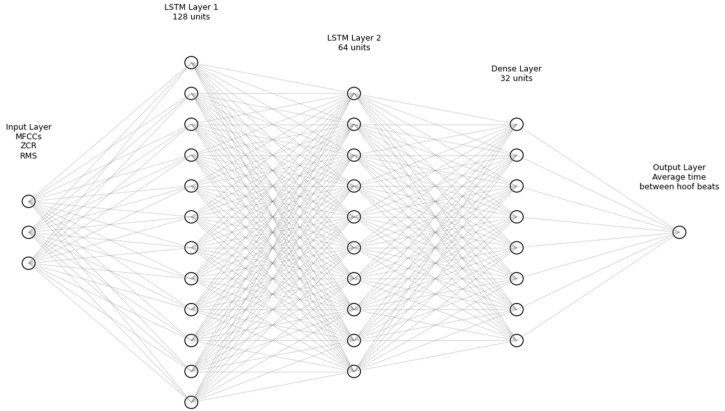
Architecture of the neural network used to predict the average time between hoof–ground contact events. The model receives acoustic features (MFCCs, RMS, ZCR) as input and processes them through two LSTM layers followed by a dense layer, producing a single regression output.

The architecture of the neural network implemented in this study is demonstrated in Figure 1. The model is composed of a sequence of layers specifically designed to process time-series acoustic data. The input layer receives a matrix of temporal acoustic features, including Mel-frequency cepstral coefficients (MFCCs), root mean square energy (RMS), and zero-crossing rate (ZCR), extracted from audio recordings of hoof–ground contact events. These features are fed into two stacked long short-term memory (LSTM) layers with 128 and 64 units, respectively. LSTM units are capable of capturing temporal dependencies within sequential data by maintaining internal memory states across time steps. Each LSTM layer is followed by a dropout layer (30%) to reduce the risk of overfitting. After the LSTM blocks, a fully connected dense layer with 32 units processes the output sequence, followed by a final dense layer with a single unit that produces the predicted average interval between successive hoof beats. This architecture allows the model to learn both the temporal dynamics and the spectral patterns embedded in the audio signal, making it suitable for gait parameter estimation based solely on hoof sound recordings.

### 2.7. Deep Learning Model Training

The deep learning model, based on the long short-term memory (LSTM) architecture, underwent a supervised training process aimed at predicting the time intervals between successive hoof support phases in equines. The training was conducted over 100 epochs, using a batch size of 16, which allowed for efficient weight updates in each iteration. To minimize prediction errors, the mean squared error (MSE) loss function was used, aiming to reduce the mean squared error between predicted and actual values. This loss function is widely applied in regression problems, as it penalizes larger deviations more intensely, helping the model improve its predictions [45]. Network weight adjustments were performed using the Adam optimizer, known for combining the advantages of stochastic gradient descent with momentum and RMSProp, ensuring faster and more accurate convergence [46]. The learning rate was set at 0.001, balancing learning speed and model stability [47].

The dataset was then stratified, allocating 80% of the samples for training and 20% for testing, ensuring that the model was evaluated on previously unseen data. This data split enabled a fair assessment of the model’s ability to generalize to new data, preventing overestimation of performance due to reliance on a specific sample set [27].

### 2.8. Cross-Validation (K-Fold)

To ensure that the model exhibited consistent performance across different data subdivisions, the k-fold cross-validation technique was applied, using 5 folds. This method consists of dividing the dataset into five equal parts. In each iteration, four parts are used for training and one for validation, ensuring that all parts serve as a validation set at some point. This process reduces the model’s dependency on a single data split, providing greater reliability in performance assessment [48,49].

During each iteration of cross-validation, the model’s performance was assessed using three evaluation metrics. For mean absolute error (MAE) [43,44], the computation involved the absolute difference between the predicted value $\left({\hat{y}}_{j}^{\left(i\right)}\right)$ and the actual value $\left(y_{j}^{\left(i\right)}\right)$ for each sample j in fold i. Root mean squared error (RMSE) [43,44] was calculated similarly but emphasized larger deviations by squaring the differences before averaging. For both metrics, the number of samples in each fold ${(N}_{i})$ and the total number of folds $\left(K\right)$ were used to compute the average. The coefficient of determination (R^2^) [37,38] evaluated how well the predicted values approximated the actual values, based on the variance within each fold. In this case, the mean of the observed values $\left(y_{j}^{\left(i\right)}\right)$ was also incorporated into the calculation to assess the proportion of explained variance.

In addition to the MSE, MAE, RMSE, and R^2^ metrics, the mean absolute percentage error (MAPE) [43,44] was also computed. This metric expresses the prediction error as a percentage of the actual values. Specifically, it uses the absolute difference between the predicted value $\left({\hat{y}}_{i}\right)$ and the actual value ($y_{i}$), divided by ${(y}_{i})$, and averaged across all $\left(N\right)$ samples. The MAPE provides an intuitive measure of model accuracy, with lower values indicating better predictive performance.

Cross-validation allowed for the evaluation of the model’s accuracy as well as its robustness and generalization capability, ensuring that it performed consistently across different data subsets. This procedure is essential to prevent the model from displaying overly optimistic performance on a single test set, ensuring a more comprehensive and reliable evaluation.

### 2.9. Calculation of the Degree of Dissociation

After the model validation, the predicted time intervals between hoof support phases were used to calculate the dissociation, a fundamental metric in the analysis of equine gaits. This calculation was performed by considering the relative differences between the time intervals of the four hooves’ support phases in each complete gait cycle, allowing for the identification of synchronization or desynchronization levels between hoof–ground contacts.

The dissociation (in % of the complete audio) was calculated using the following formula:

(1) $$\mathrm{Dissociation}\;(\%)\;=\;\left(1-\;\frac{\sum_{i=1}^{N-1}\left|\frac{{interval}_{i+1}-{interval}_{i}}{{interval}_{i}}\right|}{N-1}\right)\;\times \;100$$

In this expression, *N* represents the total number of hoof supports in a gait cycle, while interval*_i_* is the time between one hoof–ground contact and the next. The summation (Σ) calculates the sum of the relative differences between consecutive intervals, and this average is normalized by the total number of differences (*N* − 1), representing the regularity of the gait cycle.

This calculation enables the analysis of variations within each gait cycle, considering temporal nuances in the hoof–ground contact sequence. Each gait cycle is processed individually, and the mean dissociation value across all cycles is recorded as the final result for each analyzed audio sample.

### 2.10. Statistical Analysis

All statistical analyses were performed using JMP Pro version 18.2 (SAS Institute Inc., Cary, NC, USA). We assessed the resulting variable normality through a Shapiro–Wilks test. For non-normally distributed data, we applied a non-parametric statistical approach (Wilcoxon/Kruskall–Wallis) with 95% confidence intervals to evaluate the associations between variables, as well as mean time between hoofbeats and dissociation degree, with gait type (marcha batida and marcha picada) and breed (Campolina, Piquira, and Mangalarga Marchador), whereas statistical significance was established at *p* ≤ 0.05 and trend towards significance if *p* ≤ 0.10.

To facilitate biomechanical interpretation, the acoustic features were grouped and renamed according to their expected biological significance. RMS was interpreted as sound intensity, reflecting the amplitude of hoof–ground impacts. ZCR was associated with sound tone, indicating the frequency of signal oscillations around the zero axis, which is relevant for characterizing gait rhythm. MFCC 1 was treated as an individual descriptor of fundamental tone, while MFCCs 2 to 5 were grouped under “shape,” describing the overall contour of the frequency spectrum. MFCCs 6 to 13 were categorized as “sound components”, representing finer-grained spectral information potentially linked to subtleties in hoof–ground contact patterns and ground surface interaction.

To explore the structure and relevance of the acoustic variables extracted from the audio signals, specifically root mean square energy (RMS), zero crossing rate (ZCR), and Mel-frequency cepstral coefficients (MFCCs 1–13), a covariance principal component analysis (PCA) was performed. The objective of this PCA was not dimensionality reduction but rather the evaluation of the variance structure of the dataset, ensuring that the extracted features captured biologically meaningful distinctions among gait types and breeds.

## 3. Results

### 3.1. Audio Quality and Effectiveness of Pre-Processing

**Figure 2:**
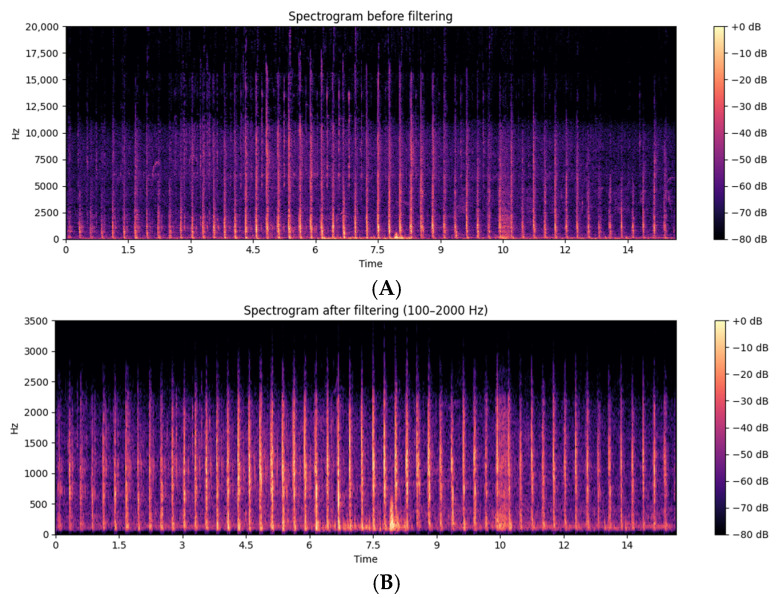
Spectrograms of random audio samples with noise (**A**) and without noise (**B**).

The noise reduction process resulted in a visual difference between spectrograms of audio signals (Figure 2). The post-processed audio files exhibited clearly defined frequency bands over time, with less dispersion of low-amplitude components. In contrast, the spectrogram of the original audio with noise shows greater frequency dispersion and the presence of low-intensity components distributed throughout the spectrum.

**Figure 3:**
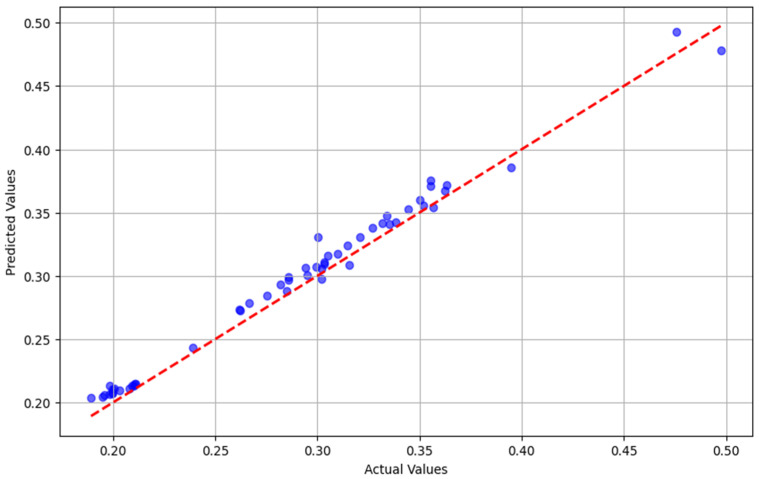
Scatter plot of predicted vs. actual values for the LSTM model demonstrating the R2 correlation of 0.98 between model-generated values (predicted) versus observed values within the dataset.

The effectiveness of the audio processing, with an accuracy of R^2^ = 0.98, can be due to the robust performance of the artificial neural network model (Table 1, Figure 3), reinforcing its accuracy in handling real-world data.

### 3.2. Performance of the LSTM Model

**Figure 4:**
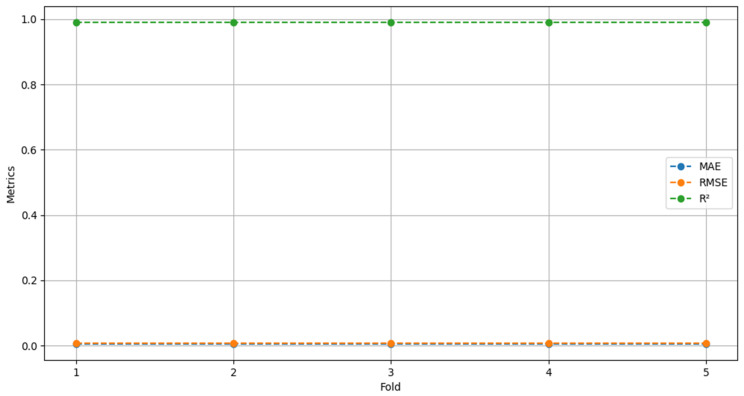
Cross-validation by fold for the LSTM model.

The results of the five-fold cross-validation showed no significant variations that could compromise the robustness of the LSTM model in predicting hoof–ground contact moments (Figure 4). This suggests that the model’s performance remained stable despite the data being partitioned into different subsets during cross-validation.

Model performance across the five-fold cross-validation, considering R^2^, RMSE, and MAE metrics. R^2^ remained consistently close to 1, while RMSE and MAE showed values very close to zero. For this reason, the blue (MAE) and orange (RMSE) lines are not clearly visible in the plot.

### 3.3. Hoof–Ground Contact Patterns

**Figure 5:**
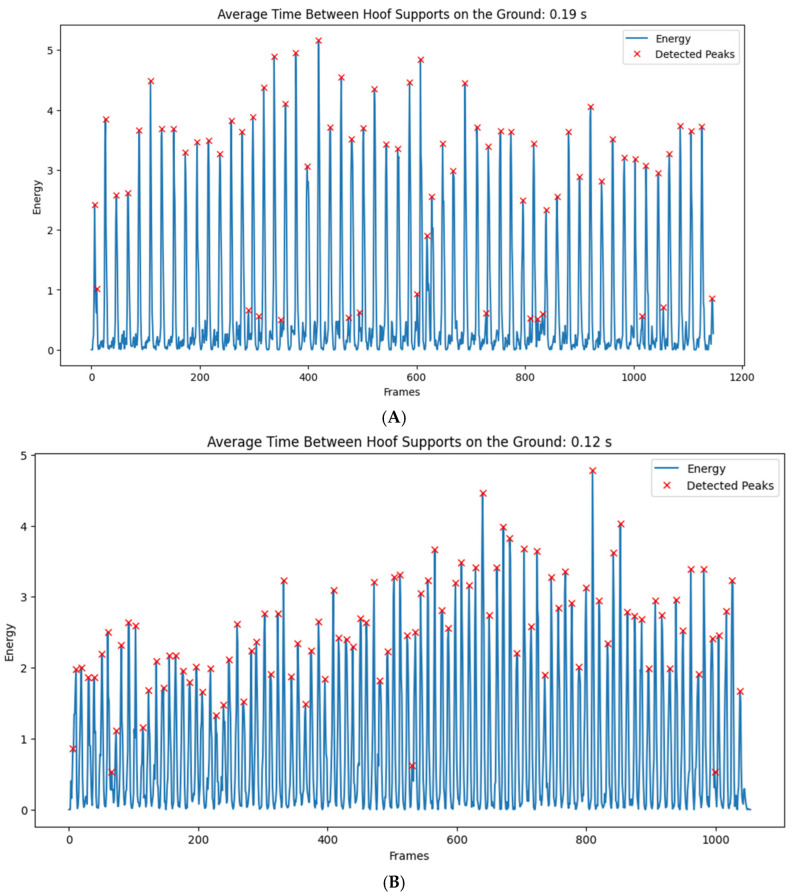
Detected moments (peaks) of hoof–ground contact for (**A**) marcha batida and (**B**) marcha picada. The *X*-axis represents frames (analysis windows; each frame ≈ 11.6 milliseconds at 44.1 kHz, hop_length = 512), while the *Y*-axis represents normalized energy (arbitrary units).

The predictions regarding the detected moments (peaks) of hoof–ground contact for the marcha batida and marcha picada patterns over time demonstrate rhythmic variations likely related to individual differences among the animals and the type of gait (marcha batida or marcha picada) (Figure 5).

### 3.4. Effects of Breed and Gait Type

**Figure 6:**
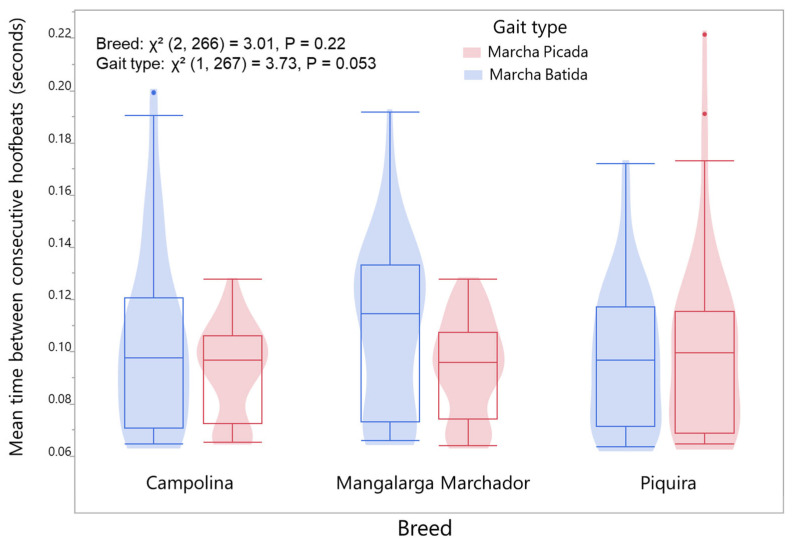
Distribution of the mean time interval between successive hoof–ground contacts across breeds and gait types, with 95% confidence intervals. Values are expressed in seconds.

**Figure 7:**
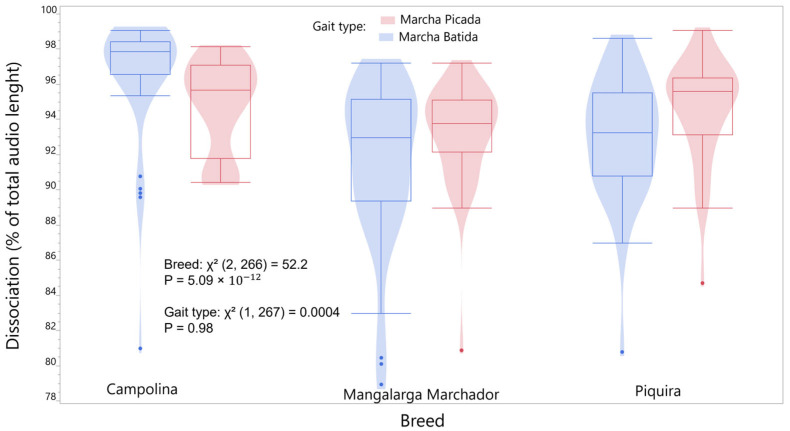
Distribution of dissociation (%) across breeds and gait types, with 95% confidence intervals.

Gait type (Χ^2^ (1, 267) = 3.73, *p* = 0.053) but not breed demonstrated a trend towards significance in association with the average time between successive hoof–ground contact (Figure 6). In contrast, we observed a strong association between breed and dissociation percentage (Χ^2^ (2, 266) = 52.2, *p* = 5.09 × 10^−12^), whereas gait type did not have a significant relationship (Figure 7).

### 3.5. Multivariate Structure of Acoustic Descriptors

**Figure 8:**
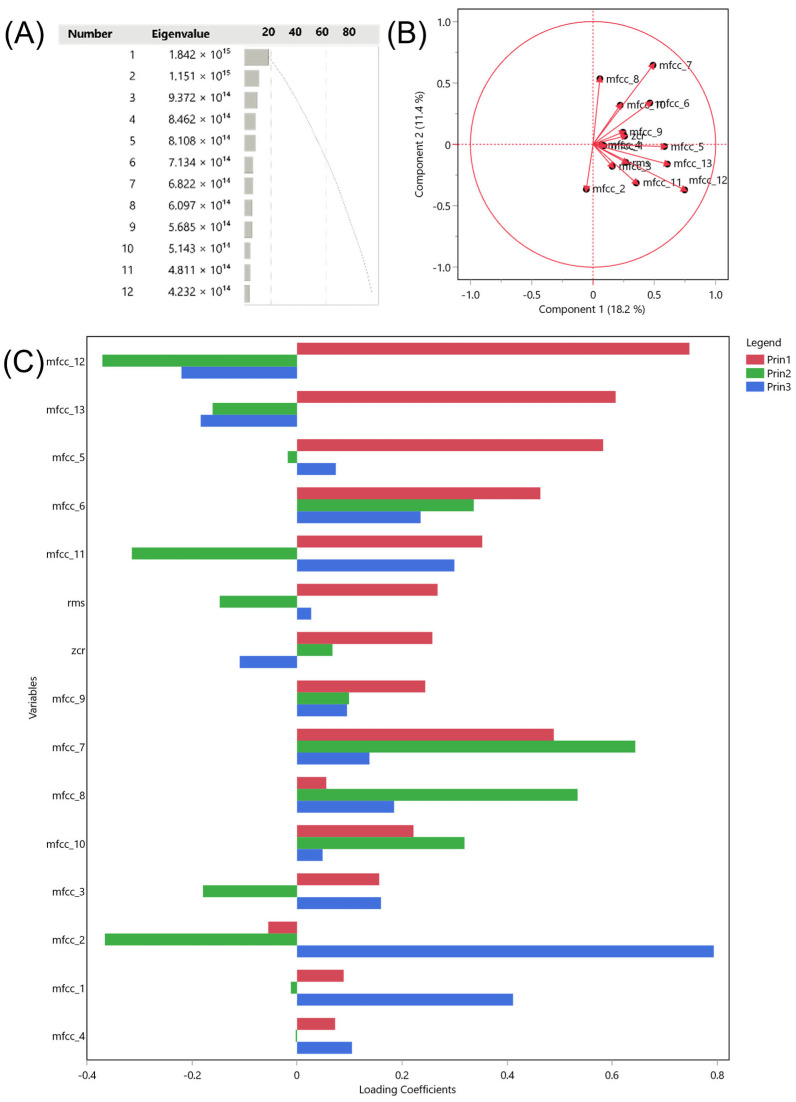
Covariance principal component analysis (PCA) of biologically grouped acoustic variables: (**A**) scree plot of eigenvalues; (**B**) correlation circle showing variable loadings on PC1 and PC2; and (**C**) partial contributions of each variable to PC1, PC2, and PC3.

The first ten components accounted for over 80% of the total covariance of the dataset, with PC1 representing 18.2% and PC2, 11.4%. Variables related to sound components and shape had a stronger influence on the construction of PC1 and PC2. In contrast, variables representing sound intensity and tone had a lower contribution to the first two PCs and were mainly represented by PC3 (Figure 8).

**Figure 9:**
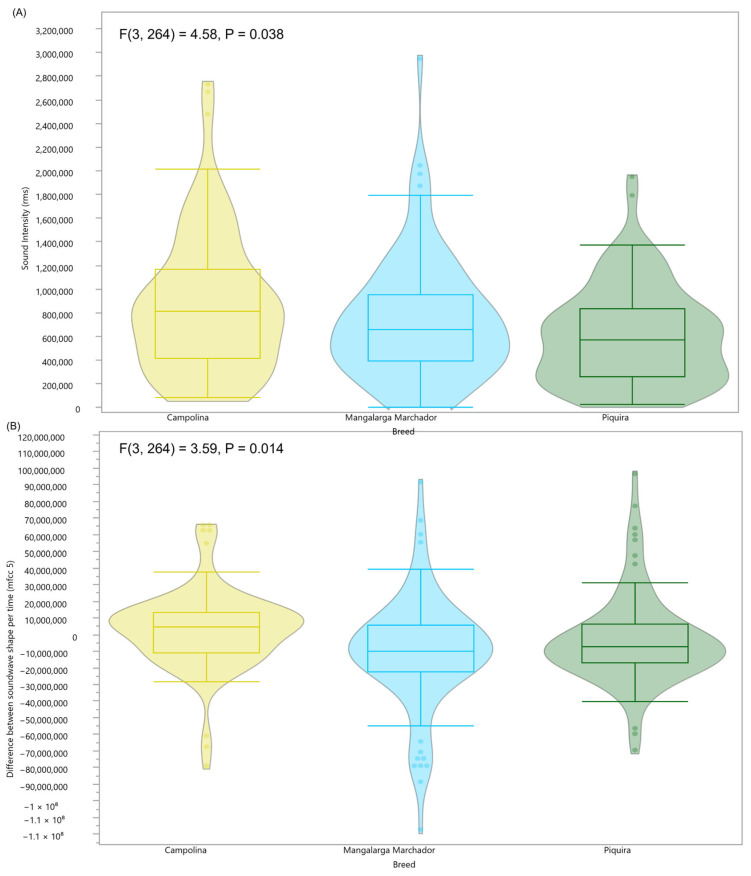

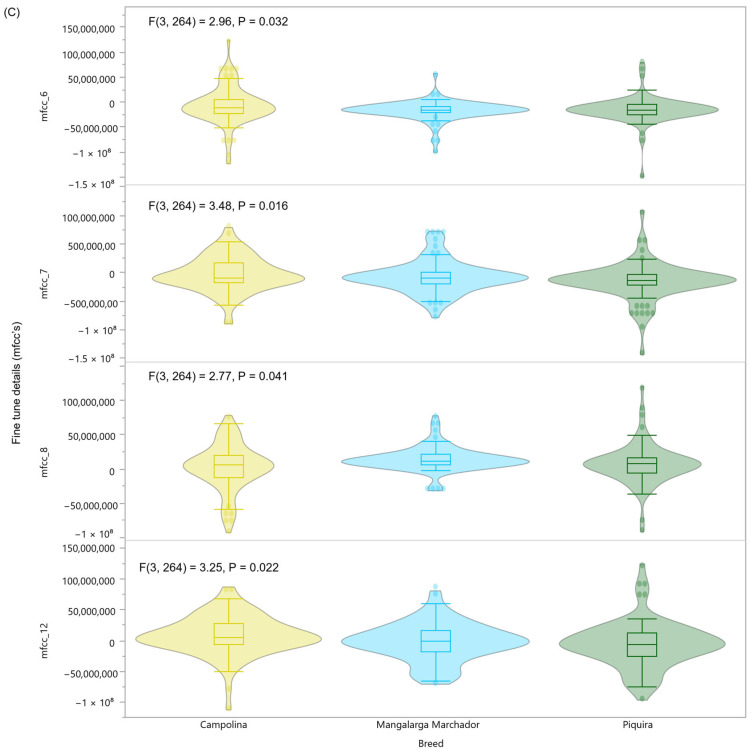
Violin boxplots of the sound spectral features significantly associated with breed, where (**A**) represents the feature related to sound intensity, (**B**) represents the feature related to the difference between the soundwave shape per time, and (**C**) represents the four features related to the fine-tuned details. (Yellow = Campolina, Blue = Mangalarga Marchador, and Green = Piquira).

There were significant differences in the decomposition of acoustic components among the evaluated breeds, with the Campolina showing the highest mean sound intensity (F(3, 264) = 4.58, *p* = 0.038) and difference between the sound wave shape per time (F(3, 264) = 3.59, *p* = 0.014). Breeds also significantly influenced the fine-tuned details (mfcc_6, 7, 8, and 12) derived from the sound spectral features (Figure 9).

**Figure 10:**
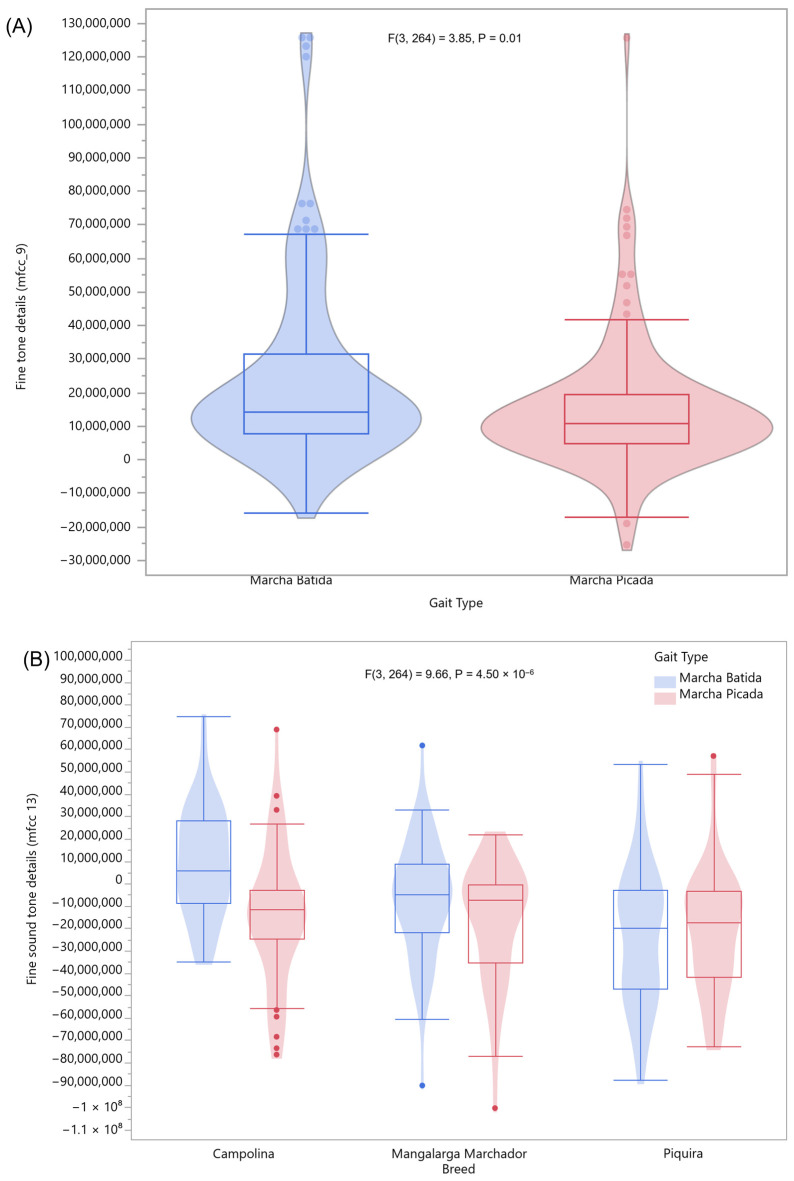
Violin boxplots of the sound spectral features significantly associated with gait type or breed, where (**A**) represents the feature related to fine tone details (mfcc_9) and (**B**) represents the feature related to fine sound tone details (mfcc_13). (Red = marcha picada, Blue = marcha batida).

Two acoustic features were also significantly associated with the four-beat gait type as categorized by the trained observer (mfcc_9) and with breed and gait type (mfcc_13), whereas the marcha batida, especially when performed by Campolina horses, demonstrates a higher mean fine tone details value (Figure 10).

Additional results and complete output tables are available in the Supplementary Materials (Dataset S1).

## 4. Discussion

Historically, in Brazil, gait analysis has focused on subjective evaluations or expensive and complex technologies such as optical systems and inertial sensors [50]. However, these methods can face limitations that restrict their large-scale practical application. Our results demonstrate that biomechanically relevant information regarding hoof dissociation and unique aspects related to horse breed and gait type can be extracted from audio-only files of Brazilian equine breeds. By utilizing a robust recurrent neural network model with long short-term memory (LSTM) units, trained with Mel-frequency cepstral coefficients (MFCCs), waveform energy (RMSE), and zero-crossing rate (ZCR), we achieved high predictive accuracy (R^2^ = 0.98). While preprocessing techniques were essential to ensure the quality of the analyzed data, especially as the audio files were collected from freely available and not standardized online resources, data augmentation allowed the model to be trained on a more robust and diverse dataset, increasing its generalization capacity across different scenarios [33]. Although the LSTM model was initially developed to estimate the intervals between hoofbeats, results suggest that the acoustic data captured sufficiently consistent patterns to distinguish, albeit unintentionally, both the type of gait and the breed of the horses (Figure 9 and Figure 10). It is possible that audio files collected utilizing a standardized methodology achieve a higher ability to differentiate dissociation percentage, gait type, and breed; thus, a larger study implementing specialized audio sampling is recommended.

### 4.1. Functional Interpretation of Acoustic Features

Variables such as sound intensity (RMS), noise uniqueness (ZCR), and MFCCs, all reinterpreted with functional biological value, likely carry relevant locomotor signals. Assigning functional names to sound spectral features associates spectral–temporal acoustic patterns with observable biomechanical properties such as limb support intensity, movement regularity, and hoof impact uniqueness [51,52]. Future analysis should aim to compare these features to unique kinematic phenotypes. However, the observed results may also reflect uncontrolled factors such as recording environment, background noise, or rider/environmental influences. Additionally, the absence of synchronized biomechanical validation, as well as the inability of the PCA to produce clusters of features associated with gait type or breed, prevents any conclusive claims about classification power. Nevertheless, the results represent a relevant exploratory finding, suggesting that functionally interpreted acoustic variables may serve as promising objective biomarkers for distinguishing gait patterns and breeds, an aspect not previously reported [27].

The analysis of limb dissociation revealed no significant difference between marcha batida and marcha picada; however, the resulting dissociation levels suggest that these gait types are in fact four-beat dissociated gaits, with each hoof contacting the ground at distinct times. While our data does not allow for individual limb dissociation ascertainment, in the future, the dissociation percentage could be calculated in more detail across different support types (lateral, diagonal, and triple), opening new perspectives for understanding gait patterns. This kinematic variation is essential in marcha picada and marcha batida, which differ from gaits such as the trot, where complete suspension phases occur [53]. Furthermore, it is possible that the order and duration of temporal intervals between hoof sounds during locomotion may reflect the predominant type of dissociation (lateral or diagonal) characteristic of each gait [9,54,55].

In marcha picada, where the bipedal lateral limb support predominates, the shortest time intervals between consecutive hoof sounds would be associated with ipsilateral limb support (forelimb and hindlimb on the same side), whereas the longer intervals would correspond to diagonal supports, and the intermediate intervals could indicate triple support moments. In marcha batida, which features diagonal predominance, this pattern would be reversed, with shorter intervals indicating diagonal support and longer intervals indicating lateral support, which aligns with videographic findings [9]. This is also consistent with the biomechanical description of limb support sequences in the Mangalarga Marchador horses performing the marcha batida, where the shortest intervals between successive supports occurred during diagonal support or diagonal dissociation [55,56]. However, this inference requires further validation when utilizing audio-only analysis. Synchronization between acoustic data and locomotion pattern kinematics would allow validation of whether shorter and longer time intervals between hoofbeats indeed correspond to lateral and diagonal supports.

### 4.2. Breed Differences in Dissociation

Among breeds, Campolina horses showed the highest mean dissociation values. Although this finding aligns with empirical observations and breed standards that indicate a greater stride amplitude in this breed [5], the literature lacks consensus regarding the direct association between morphological conformation and acoustic dissociation. The greater wither height (1.62 m for males and 1.56 m for females in Campolina; 1.50 m for males and 1.46 m for females in Mangalarga Marchador; 1.22 m for males and 1.20 m for females in Piquira), along with likely longer limb length and the typical hindlimb movement pattern observed in Campolina horses, could contribute to a longer flight time (e.g., when a given limb is not in contact with the ground or in stance/support moment) between limbs, which would result in a longer landing interval between hooves, and thus, a greater interval between successive hoof contacts [4,5,6]. However, this inference still lacks direct biomechanical validation using standardized morphokinetic data [57]. Nevertheless, the observed differences could represent biological gait variation or instead reflect variations induced by sampling, environmental conditions, or riding technique.

### 4.3. Insights from PCA and Acoustic Features

The principal component analysis (PCA) enabled a visualization of the multivariate structure of the acoustic data, with the first two components explaining approximately 29.6% of the total covariance in the dataset, and 10 PCs recapitulating over 80% of the total variance. While PCA was not able to differentiate breed and gait type, our results suggest that the acoustic descriptors may have functional discriminative potential. However, a cautious interpretation is warranted: the separation observed in the PCA, while promising, remains exploratory and may be influenced by extrinsic factors such as surface type, acoustic environment, and recording distance [58,59].

The variables sound intensity and noise uniqueness, although named based on functional interpretations, have conceptual support in biomechanics. Recent studies demonstrate that inertial sensors can accurately detect hoof contact and lift-off events, establishing clear relationships between impact intensity, surface type, and movement patterns [60]. Based on our findings, the acoustic intensity captured in the audio recordings serves as a functional proxy for impact force, consistent with previous reports of a deep learning model accurately predicting impact parameters, including force, based on head kinematics during helmet impacts in humans [61]. As for spectral entropy indexes, these may reflect variations in the regularity of limb support patterns, in alignment with reports of the use of biomechanical features to detect head impacts [62].

### 4.4. Methodological Constraints and Future Research Needs

Previous studies in human gait acoustics have demonstrated that multiple variables, including footwear, ground surface, and locomotor fatigue, influence both the amplitude and spectral content of footstep sounds [20,63,64,65]. Comparable effects are expected in equids. For example, hoof shoeing and trimming techniques are known to alter hoof–ground impact dynamics and loading symmetry, while surface type and texture affect ground reaction forces, vibration transmission, and, consequently, the acoustic signal. Training background also influences locomotor regularity and coordination, thereby shaping rhythmicity and temporal dissociation patterns. In the present study, we were unable to control these factors because our recordings were extracted from publicly available online sources. Future studies should aim to acquire data under controlled conditions with standardized surfaces, consistent hoof trimming or shoeing status, and trained versus untrained subgroups, thereby minimizing environmental confounding factors. Algorithmic approaches such as signal normalization, domain adaptation, and noise-robust feature extraction may further mitigate these sources of variability.

An additional limitation is the absence of an in-person veterinary verification of animal soundness. Low-grade lameness or subtle asymmetry, even when not readily apparent, can significantly alter stride dynamics and event timing, and this has been repeatedly documented in both equine gait analysis and human acoustic gait studies. In our dataset, despite no clear evident lameness or asymmetry, horses may have varied in their locomotor health or fatigue state, which could affect the robustness of dissociation measures estimated from audio. We therefore emphasize that future controlled studies should include clinical soundness examinations, objective lameness screening (e.g., via inertial sensor–based symmetry indices), and fatigue monitoring to establish reliable baselines. The absence of a control group is another limitation. In future experiments, we recommend incorporating known conditions, such as sound horses compared with horses exhibiting induced gait asymmetry or experimentally fatigued horses, to enable direct benchmarking of acoustic signatures.

An important technical consideration relates to the acoustic devices themselves. Prior work in human gait acoustics has shown that microphone type, placement, and recording environment contribute substantial variability, including latency shifts in detecting foot–ground contact [63,64,65]. In the present study, recordings were obtained from heterogeneous video sources with uncontrolled device settings, likely contributing to residual variation. This contrasts with controlled laboratory environments, in which microphone arrays and calibrated devices can substantially improve consistency and spatiotemporal accuracy. Future equine-focused studies should adopt standardized recording protocols, including fixed microphone positioning relative to the arena, known sampling rates, and calibration routines to ensure repeatability.

Furthermore, the dataset was derived from public videos with heterogeneous audio quality and environmental noise, which may have affected signal consistency. While our preprocessing pipeline mitigated most artifacts, controlled standardized recordings under controlled conditions would further improve measurement precision. Second, breed and gait annotations were assigned by a single expert evaluator, introducing potential labeling bias. Third, the absence of ground truth validation using kinetic or sensor-based reference systems limits the validation of the dissociation measures inferred from sound. Lastly, although the LSTM model performed well under the given conditions, its generalization to other breeds, sampling rates, or untrained conditions still needs to be validated.

Despite these limitations, the present study represents a conceptual advance in quantifying equine intermediate gaits using acoustic data. From an applied perspective, this approach provides a potential low-cost, non-invasive complement to current gait analysis technologies. Successful large-scale application will require standardized recording protocols, multicenter validation, and user-friendly interfaces that can be deployed reliably by field technicians and breed inspectors. Future work should emphasize controlled data acquisition with verified pedigrees and multi-evaluator classifications while also exploring multimodal approaches that combine acoustic signals with video, inertial sensors, or force platforms to capture a more comprehensive picture of gait phenotypes. Advances in deep learning, particularly temporal attention models and contrastive self-supervised learning, have the potential for improving robustness, interpretability, and the capacity to model real-world variation in animal husbandry. Embedding such audio-based models into breed evaluation protocols would represent a significant step toward digital phenotyping in animal science, enabling objective, scalable, and standardized metrics to replace or complement subjective sensory assessments in gaited horses.

## 5. Conclusions

The present study demonstrates that acoustic analysis of hoof–ground contact sounds, processed with spectral and temporal features (MFCCs, RMS, ZCR) and modeled through a long short-term memory (LSTM) neural network, can accurately quantify dissociation timing in Brazilian gaited horses. The model achieved high predictive performance (R^2^ = 0.98, MAPE = 2.04%), confirming the feasibility of using audio-only data to extract biomechanical parameters relevant to equine gait evaluation.

While dissociation percentage did not differ significantly between marcha batida and marcha picada, the results confirmed that both are distinct four-beat dissociated gaits. Breed differences were evident, with Campolina horses showing longer dissociation intervals, suggesting that acoustic-derived dissociation measures may capture biologically relevant locomotor variation. Principal component analysis further indicated that multivariate acoustic descriptors encapsulate meaningful biomechanical information, even though clear clustering of gait type or breed was not achieved. Together, these findings provide proof-of-concept that standardized acoustic recordings can yield objective and quantifiable insights into equine intermediate gaits. This study demonstrates, for the first time, that hoof impact sounds contain sufficient information to distinguish biomechanically relevant features of gait type and breed, highlighting their potential as low-cost, non-invasive indicators for use in practical evaluation contexts.

## Figures and Tables

**Table 1:** LSTM model metrics for the correlation between observed and predicted values of the dataset.

Mean Absolute Error (MAE)	Mean Squared Error (MSE)	R^2^ Score	Root Mean Squared Error (RMSE)	Mean Absolute Percentage Error (MAPE)
0.0071	0.0001	0.98	0.0078	2.04%

## Data Availability

Data will be made available upon request to the corresponding authors of this study.

## Supplementary Materials

The following supporting information can be downloaded at: https://www.mdpi.com/article/10.3390/ani16091283/s1, Table S1: Extracted audio features used in the statistical analyses (supplementary material (features).csv); Table S2: Additional statistical results of the analyses (supplementary material results.csv); Dataset S1: Augmented audio files of Campolina, Mangalarga Marchador, and Piquira horses, available at: https://doi.org/10.5281/zenodo.15863180 (accessed on 28 August 2025).
